# The Effect of Betulin Diphosphate in Wound Dressings of Bacterial Cellulose-ZnO NPs on Platelet Aggregation and the Activity of Oxidoreductases Regulated by NAD(P)+/NAD(P)H-Balance in Burns on Rats

**DOI:** 10.3390/molecules26185478

**Published:** 2021-09-09

**Authors:** Nina Melnikova, Darina Malygina, Alyona Balakireva, Peter Peretyagin, Vadim Revin, Anna Devyataeva, Kseniya Malafeeva, Viktor Revin

**Affiliations:** 1 Faculty of Chemistry, Lobachevsky University, 23/5 Gagarin Av., 603950 Nizhny Novgorod, Russia; 2 Department of Pharmaceutical Chemistry, Privolzhsky Research Medical University, 10/1 Minin Sq., 603950 Nizhny Novgorod, Russia; mds73@yandex.ru; 3 Central Research Laboratory, Privolzhsky Research Medical University, 10/1 Minin Sq., 603950 Nizhny Novgorod, Russia; Liza200000@yandex.ru (A.B.); peretyaginpv@gmail.com (P.P.); 4 Department of Biotechnology, Bioengineering and Biochemistry, National Research Ogarev Mordovia State University, 68 Bolshevistskaya Str., 430005 Saransk, Russia; vadim.revin16@mail.ru (V.R.); ania.devyataeva@yandex.ru (A.D.); ksenya.malafeeva@yandex.ru (K.M.); revinvv2010@yandex.ru (V.R.)

**Keywords:** betulin diphosphate, wound dressings, ZnO NPs, bacterial cellulose, anti-aggregation, oxidoreductase activity

## Abstract

The inhibition of platelet aggregation, and the activity of oxidoreductases and microhemocirculation in a burn wound on the treatment of burns with wound dressings based on bacterial nanocellulose (BC)-zinc oxide nanoparticles (ZnO NPs)-betulin diphosphate (BDP) were studied. The control of the treatment by BC-ZnO NPs-BDP on burned rats by the noninvasive DLF method showed an increase in perfusion and the respiratory component in wavelet spectra, characterizing an improvement in oxygen saturation in the wound. The study on the volunteers’ blood found the inhibition of ADP-induced platelet aggregation by 30–90%. Disaggregation depends on the dose under the action of the ionized form of BDP and ZnO NPs-BDP in a phosphate buffer; it was reversible and had two waves. It was shown on rats that the specific activity of LDH_reverse_ and LDH_direct_ (control-intact animals) on day 21 of treatment increased by 11–38% and 23%, respectively. The LDH_reverse_/LDH_direct_ ratio increased at BC-ZnO NPs-BDP treatment, which characterizes efficient NAD+ regeneration. AlDH activity increased significantly in the first 10 days by 70–170%, reflecting the effectiveness of the enzyme and NAD+ in utilizing toxic aldehydes at this stage of burn disease. The activities of GR and G6PDH using NADP(H) were increased with BC-ZnO NPs-BDP treatment.

## 1. Introduction

Oxidative stress and a decrease in antioxidant enzymatic protection, coupled with a change in the ratio of the level of pairs of pyridine nucleotides NAD+/NADH and NADP+/NADPH, are links in the triggering mechanism of energy and redox metabolism disorders that are associated with the causes of many diseases [1,2,3,4]. To treat such serious diseases as burn disease, it is also important to understand cellular redox homeostasis and energy metabolism induced by hypoxia (hypoxia-inducible factor) [2].

NADH/NAD+ and NAD+/NADH redox imbalance are the initiators of reductive stress and oxidative stress in different diseases dealing with hypoxia. Previously, the NAD+/NADH redox state was proposed as a potential biomarker and therapeutical target, for example, in neuroprotection in glaucoma [5]. The NADH/NAD+ redox imbalance can deal with diabetic kidney disease [6]. It has been shown that circulating markers of NADH-reductive stress correlate with mitochondrial disease severity [7]. A study of burn injuries using laser Doppler and oxygen saturation, and the subsequent quantification of ATP, ADP, NAD+, and NADH by HPLC showed that severe burns change the NAD+/NADH ratio, reflecting the course of burn disease [2].

It is known that hypoxia, or more accurately, dysoxia, is often an unlikely clinical scenario in trauma treatment, yet many clinicians often treat elevated lactate as if, by definition, there is an O_2_ limitation [8,9]. A measurable increase in lactate has remained a reliable predictor of poor outcomes in the clinical setting in trauma treatment [10,11], but the mechanisms behind this have not been fully elucidated.

The role of other redox-couples NADP+/NADPH in cellular energy metabolism is important too. The level of cellular NAD(H)/NADP(H) is essential for maintaining redox homeostasis. The deficiency in these redox couples can lead to oxidative or reductive stress, depending on the redox ratio of each. Both oxidative stress and reductive stress are detrimental to normal cell functions. This dual role complicates the use of global antioxidants as rational and effective therapeutic approaches to redox stress disorders. An imbalance of these two redox couples directly affects energy metabolism. This imbalance alters NAD(H)/NADP(H)-dependent enzymes and, thus, affects their functions in regulating cellular metabolism [4].

The destruction of burned tissue and damage to the epithelium lead to a disturbance in the microcirculation system, changes in the blood flow rate and the rheological properties of the blood, and a disturbance in the blood coagulation system, which enhances hypoxia [12,13].

Triterpenoids, including betulinic acid and its derivatives, exhibit antioxidant, anti-inflammatory, antiaggregatory, wound healing, and other properties necessary to treat burn wounds [14,15,16,17,18,19]. The low bioavailability and poor solubility of this class of compounds are serious obstacles to creating new dosage forms. One of the ways to improve the bioavailability of triterpenoids is their functionalization with polar groups, which increases their solubility. The modification of these compounds with phosphate and phosphoryl groups, which play important roles in metabolic reactions, makes it possible to consider the phosphoric or phosphonic acid esters of triterpenoids (both by alcohol groups and by terminal double bond) as promising active pharmaceutical ingredients (APIs) [20,21,22,23,24,25,26,27].

Earlier, in an experiment on rats, we showed that betulin diphosphate is a promising API for treating burns as a component of wound dressings made of bacterial cellulose and zinc oxide nanoparticles, and various dispersions, including oleogels with vegetable oils [28,29].

The absence of lignins and hemicelluloses, the high hydrophilicity and biocompatibility with various body tissues, the high sorption capacity concerning biological fluids, and the low toxicity make it possible to isolate bacterial cellulose as the most promising basis for the introduction of betulin derivatives.

The choice of zinc oxide nanoparticles as a vector for the delivery of triterpenoids is due to the combination of its pharmacological properties, such as bactericidal, immunomodulatory, and high skin permeability [30,31,32,33]. The most commonly used modified nanoparticles of silver and gold have shown effective bactericidal and antitumor properties, inducing bone growth, as well as other agents [34,35,36,37].

The introduction of zinc oxide nanoparticles into BC, exhibiting antioxidant and prooxidant properties, allows the regulation of the redox imbalance of the pairs of pyridine nucleotides NAD+/NADH and NADP+/NADPH, which occurs during the course of burn disease. At the same time, the redox imbalance affects the level of oxidoreductases that determine energy metabolism. In addition, the modification of either BC or ZnO NPs with betulin diphosphate can impart new properties to the composite, such as anti-aggregation properties. It is known that betulinic acid, asiatic acid, and its glycosides, amaranth triterpenoids α- and β-amyrin, can inhibit platelet aggregation induced by the adenosine 5′-diphosphate (ADP) and other agonists [38,39,40,41,42].

In this work, we continued the study of the biological properties of wound dressings for the treatment of burn wounds based on bacterial nanocellulose-zinc oxide nanoparticles-betulin diphosphate. We studied: (i) microcirculation in a burn wound using a noninvasive method of laser Doppler flowmetry; (ii) the ability of betulin diphosphate to inhibit the platelets aggregation induced by ADP using in vitro experiments on human blood; (iii) the activity of oxidoreductases coupled with the redox balance of the pair of coenzymes NAD+/NADH and the level of NADPH in experiments on rats.

## 2. Results

Groups of rats with burns without treatment (Day 0 and Day 10) were used as negative controls. The study on Day 21 in the negative control groups was not carried out, because the results were unreproducible due to the death of some rats. The group treated by Oleogel ZnO NPs-BDP and the Intact group were used as positive controls. The general appearance and condition of the animals during the treatment with wound dressings BC-ZnO NPs-BDP, BC-BDP, and BC-ZnO NPs significantly improved in comparison with the group without treatment. The burnt wound image of rats after the treatment by BC-ZnO NPs-BDP was better than those of BC-BDP and BC-ZnO NPs (Figure S1). The wound area reduced on days 10 and 21 by 10.33% and 42.31%, respectively, using BC-ZnO NPs-BDP wound dressings (Table S1).

The effect of BDP having two ionizable phosphate groups on the healing of burnt wounds may be due to the following events: respiration in the wound; ADP production after severe burn leading to platelet aggregation; and NADH/NAD+ ratio, determining oxidoreductases activity.

### 2.1. Study of Microhemocirculation in a Burn Wound

Microhemocirculation in the wound before and after the burn on Day 10 in the treatment of BC-ZnO NPs-BDP and BC-ZnO NPs was studied by the noninvasive laser Doppler flowmetry (LDF). We studied the state of the flow of erythrocytes in the blood (perfusion) and tissue oxygenation in the wound, which is important for cellular respiration [43,44].

**Figure 1:**
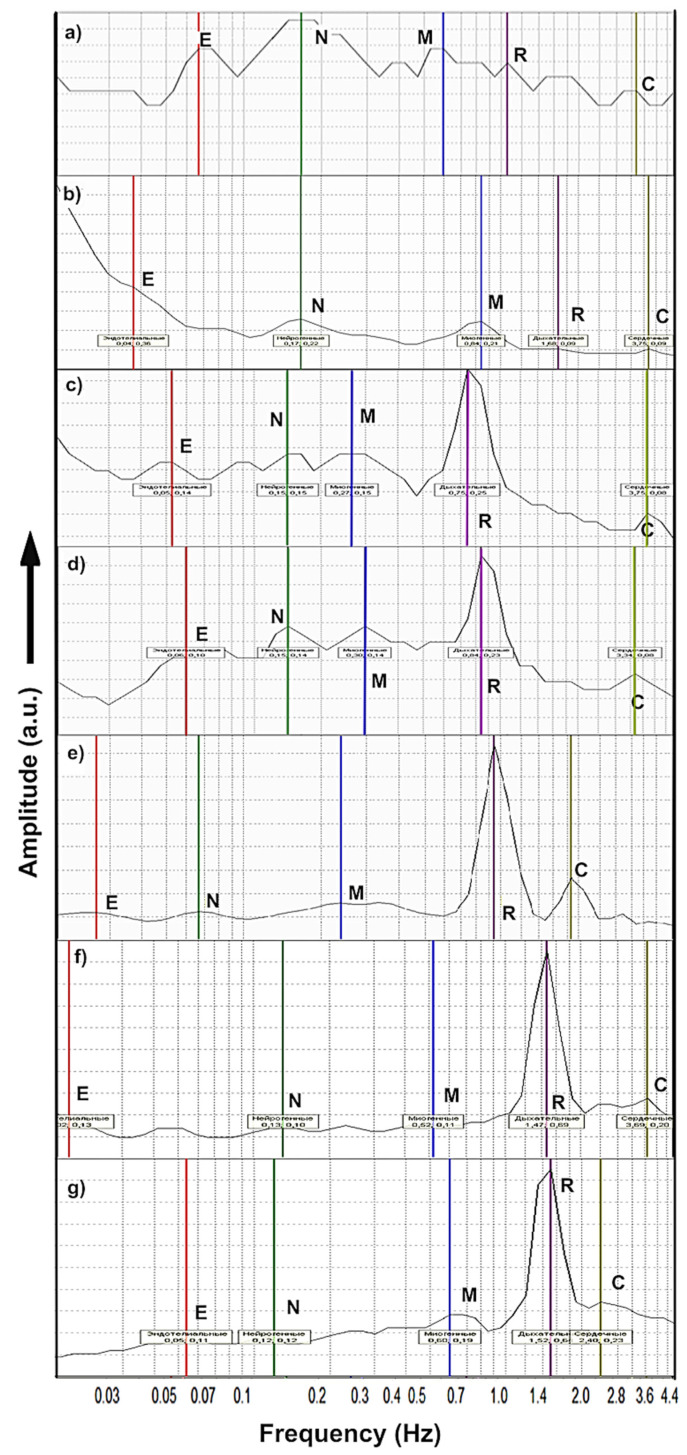
Wavelet-LDF spectra obtained immediately after a burn (Day 0 (**a**) and Day 10 (**b**)) and after treatment of burn wounds on Day 10 with: Oleogel ZnO-BDP (**c**), BC-ZnO (**d**), BC-ZnO-BDP (Day 10 (**e**) and Day 21 (**f**)). Typical spectrum of intact rats (**g**). E—endothelial, N—neurogenic, M—myogenic, R—respiratory, and C—cardiac factors.

The LDF spectra after the wavelet transform were analyzed by the approach described in [44], using the frequency regions of the Doppler reflected signal, characterizing the endothelial (E), myogenic (M), neurogenic (N), respiratory (R), and cardiological (C) components of blood circulation in a burn wound (Figure 1).

Microhemocirculation spectra of the Intact group during the experiment (21 days) were not changed, because the conditions of keeping the animals (23 ± 2 °C, air humidity 50 ± 10%, ventilation mode 30 min/h, 12/12 h lighting rhythm) were constant. Changes in the microcirculation index in animals without trauma were within the physiological norm (13.26 ± 1.21 perf. un.).

The most changes in the wavelet-DLF spectra (amplitude) during the treatment by BC-ZnO NPs and BC-ZnO NPs-BDP occurred in the frequency range of 0.5–4.5 Hz, reflecting the respiratory component R of microhemocirculation as a factor of oxygen saturation. Endothelial E, neurogenic N, and myogenic M components of the wavelet-DLF spectra changed slightly in comparison with the Intact group (Figure 1). Microhemocirculation indexes were assessed using R/E, R/N, and R/C ratios of the intensity of the wavelet spectrum amplitude (Table 1). The ratio in the respiratory R region to the amplitude intensity in the neurogenic N and endothelial E regions (R/E and R/N) significantly increased on Day 10 of treatment by all studied dosage forms.

The big differences in the R/E, R/N, and R/C ratios in the wavelet-LDF-spectra after the treatment with BC-ZnO-BDP and Oleogel ZnO-BDP are probably due to the influence of the lipophilicity of dosage forms on oxygen saturation in the wound. Unlike the lipophilic oil medium in Oleogel, hydrophilic bacterial cellulose can retain large amounts of water, provide wound hydration and absorption of exudate, and remove unwanted products from the wound.

The perfusion as microhemocirculation indexes (MIs) caused by the flow of erythrocytes in the blood had values close to those of the MIs of healthy animals on the third day, which changed up to Day 21 insignificantly. Big changes in the wavelet LDF spectra on 10–21 days of treatment with BC-ZnO-BDP can be due to an improvement in oxygen saturation into the burn zone under the influence of BDP.

In general, the most intense microcirculation in a burn wound during treatment with BC-ZnO-BDP wound dressings can be the key to improving oxygenation in the area of a burn wound and, accordingly, accelerating its healing.

### 2.2. Study of Platelet Aggregation under Action of BDP

**Figure 2:**
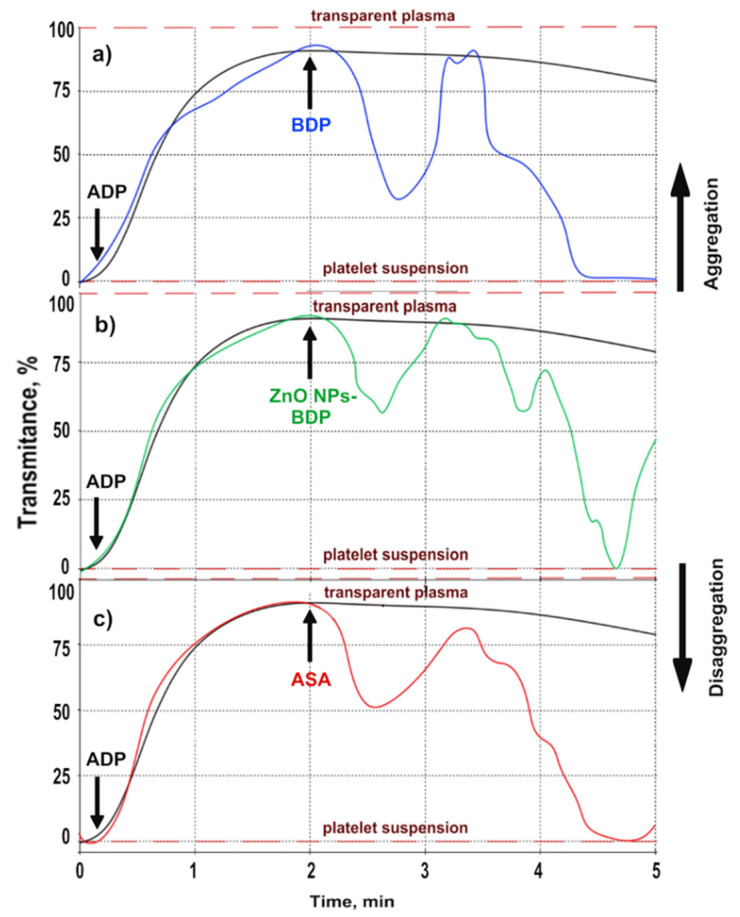
The inhibition of platelet aggregation (blood of volunteers), induced by ADP (3.75 μM), under the influence of 6.65 mM of BDP (**a**), ZnO NPs-BDP 6.65 mM (**b**), and ASA 1.40 mM (**c**). The black line is the control, which means ADP action only (3.75 μM).

**Figure 3:**
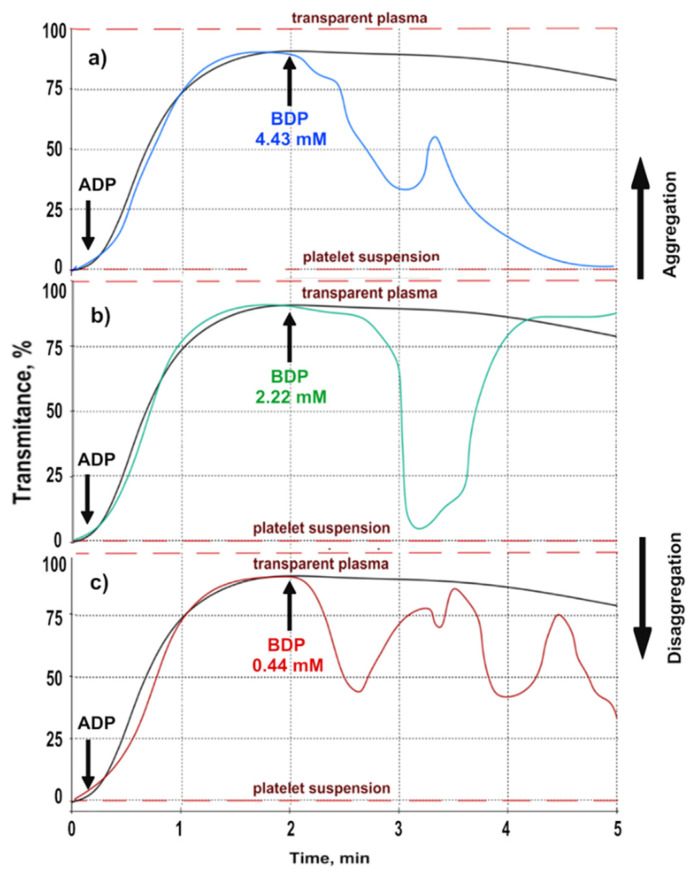
Dose-dependent inhibition of platelet aggregation (blood of volunteers) induced by ADP (3.75 μM) under the action of 4.43 mM of BDP (**a**), 2.22 mM of BDP (**b**), and 0.44 mM of BDP (**c**); black line-control, ADP action (3.75 μM).

We studied the effect of BDP on platelet aggregation in vitro in human platelet-rich plasma (PRP) activated by the agonist ADP using the turbidimetric method by the Born method [45]. Figure 2 and Figure 3 show the inhibition of platelet aggregation over time under the action of ADP and the studied substances. The black lines on Figure 2 and Figure 3 mean the control aggregation process under ADP only, without adding studied substances (BDP, ZnO NPs, their mixtures, and acetylsalicylic acid). In this case, the platelet aggregation process was the same under the constant experiment conditions. Colored lines on Figure 2 and Figure 3 show platelet aggregation under the action of the studied substances introduced after 2 min after the addition of ADP.

Acetylsalicylic acid (ASA), which exhibits both antiplatelet and anticoagulant properties, was chosen as a positive control.

The data in Figure 2 and Table 2 demonstrate the complex process of inhibition of ADP-induced platelet aggregation by 6.65 mM of BDP, ZnO NPs-BDP 6.65 mM, and acetylsalicylic acid (ASA).

In all experiments, the agonist ADP was added to platelet-rich plasma (PRP) after 10 s. In the control in the absence of inhibitors under the ADP action, platelet aggregation occurred (Figure 2a–c, black line). Aggregation inhibitors were added to PRP with ADP after two minutes. We observed the formation of a turbid solution within the next minute due to reversible platelet disaggregation. At 3.5 min (about 210 s), the second wave of platelet disaggregation began. The percent inhibition of aggregation by the addition of various agents in the presence of an ADP agonist is shown in Table 2.

The obtained data show that BDP exhibited antiaggregatory properties similar to acetylsalicylic acid. Zinc ions (ZnO NPs-BDP) reduced the inhibition of platelet aggregation twice at 2–3 min: 28% compared to 58% under the action of BDP only.

Figure 3 and Table 3 show that BDP inhibited platelet aggregation in a dose-dependent manner.

Several disaggregation waves were observed at a low BDP concentration (0.44 mM), but complete disaggregation was not achieved. At an average concentration of BDP (2.22 mM), complete aggregation was observed at the fifth minute of the experiment, with an intermediate first stage of disaggregation by almost 90%. At a high concentration of BDP (4.43 mM), stable platelet disaggregation was achieved at 5 min (complete inhibition of aggregation), while a reversible first stage of disaggregation was observed at 3 min.

Pure ZnO NPs without immobilized BDP did not have a disaggregation effect, but ZnO NPs-BDP showed the dose-dependent anti-aggregation activity.

The effect of triterpenoid concentration on platelet aggregation was studied using betulinic acid by authors [42].

### 2.3. Activity of Oxidoreductases

#### 2.3.1. LDH and AlDH-Specific Activities Controlled by Pairs of Coenzymes NAD+/NADH in Burns during Treatment with Wound Dressings Based on Bacterial Cellulose

The LDH reversible oxidation/reduction of lactate-pyruvate can be easily pushed to either lactate→pyruvate or pyruvate→lactate without the requirement for any special process or mechanism. Written in the direction of pyruvate (Pyr^−^) reduction to lactate (La^−^), the reaction is as follows:

Pyr^−^ + NADH + H^+^⇄La^−^ + NAD+.

At a pH near 7.0, the proton can be omitted.

In the direction:

Pyr^−^ + NADH→La^−^ + NAD+,

We can write the mass action ratio (MAR) of the reaction as:

$$\mathrm{MAR}=\frac{\left[{\mathrm{La}}^{-}\right]\left[\mathrm{NAD}+\right]}{\left[{\mathrm{Pyr}}^{-}\right]\left[\mathrm{NADH}\right]}$$

We suggested using the specific activity ratio (SAR) of $\frac{{\mathrm{LDH}}_{\mathrm{direct}}}{{\mathrm{LDH}}_{\mathrm{reverse}}}$ or $\frac{{\mathrm{LDH}}_{\mathrm{reverse}}}{{\mathrm{LDH}}_{\mathrm{direct}}}$ determined using [NAD+] and [NADH] concentrations and calculated by the Michaelis–Menten equation as an empirical biochemical parameter-reflected redox imbalance in this reaction.

$$\mathrm{SAR}=\frac{{\mathrm{LDH}}_{\mathrm{reverse}}}{{\mathrm{LDH}}_{\mathrm{direct}}}\;\approx f\left(\frac{\left[\mathrm{NAD}+\right]}{\left[\mathrm{NADH}\right]}\right)$$

We studied groups of intact animals, untreated animals (negative control) treated by oleogel (positive control), and groups treated by wound dressings (Table 4).

Studies have shown that the specific activity of LDH in direct and reverse reactions decreases after a burn without treatment on Day 3 and Day 7 but increases on Day 10 up to 96.43% in the direct and up to 72.72% in the reverse reaction.

On Day 3 and Day 7, the increase in LDH activity in direct and reverse reactions after treatment by Oleogel was 15–30%, compared with the untreated burn (Table 4). On the 10th and 21st days, the LDH activity in the reverse reaction returned to normal (96%), while the LDH activity in the direct reaction increased by 11% (Table 4).

Evaluation of the specific activity of LDH_reverse_ and LDH_direct_ in the treatment of wound dressings based on bacterial cellulose BC-ZnO NPs-BDP showed a better result than Oleogel (the positive control) did. On Day 21, under treatment by BC-ZnO NPs-BDP, LDH activity in the direct and reverse reactions increased by 38% and 23%, respectively, compared with intact animals. The LDH activity under the action of BC-BDP wound dressings was slightly lower than that of BC-ZnO NPs-BDP (Table 4).

In this work, we evaluated the change in the specific activity of LDH in the direct and reverse reactions, by the spectral method (λ_max_ = 340 nm for NADH) in erythrocytes (Table 4).

Table 5 shows the calculation of the $\frac{{\mathrm{LDH}}_{\mathrm{reverse}}}{{\mathrm{LDH}}_{\mathrm{direct}}}$ ratios as an empirical estimate of the correlation with the $\frac{\left[\mathrm{NAD}+\right]}{\left[\mathrm{NAADH}\right]}$ ratio using the data in Table 4.

The $\frac{{\mathrm{LDH}}_{\mathrm{reverse}}}{{\mathrm{LDH}}_{\mathrm{direct}}}$ ratio decreased in the group of burned animals without treatment, and this ratio was lower than for intact animals, which indirectly characterizes the low NAD+ regeneration in the reaction of pyruvate with NADH. This means a decrease in the concentration of NAD+ during the burn disease compared to the initial burn wound state.

Cytosolic lactate-pyruvate and NAD+/NADH ratios determined by routine assays of lactate and pyruvate in resting skeletal muscle yield a ratio (lactate/pyruvate) of about 10 [46].

LDH is a very-high-activity cytosolic enzyme with a strong advance in response to small changes in a substrate or product concentrations, and finally, small changes in the SAR.

We also studied the specific activity of the aldehyde dehydrogenase (AlDH), which is involved in many reactions in the human body. One of the most important AlDH reactions is utilizing the lipid peroxidation product malondialdehyde by the pair NAD+/NADH’s participation.

In the rats’ burn wound model, we showed that the AlDH-specific activity under treatment by the studied drugs increased compared to untreated burned animals during the initial treatment period (10 days) by 70–170% (Table 6).

On the 21st day of treatment, the AlDH level approached the level of healthy animals.

#### 2.3.2. The Study of the Activity of Glutathione Reductase and Glucose-6-Phosphate Dehydrogenase Catalyzed by a Pair of NADP+/NADPH Coenzymes

Table 7 shows data on the specific activity of glutathione reductase (GR) and glucose-6-phosphate dehydrogenase (G6PDH) by the action of the studied drugs (Oleogel ZnO NPs-BDP, BC-BDP, and BC-ZnO NPs-BDP).

On the 21st day, an increase in the level of G6PDH to 91% compared to the control was observed. An important function of this enzyme is the formation of cellular NADPH from NADP+, which is necessary to maintain the level of reduced glutathione in the cell, and the synthesis of fatty acids and isoprenoids.

## 3. Discussion

The increase in the respiratory component’s amplitude in wavelet-LDF spectra and in microhemocirculation indexes (Table 1, Figure 1) confirmed an intensification of oxygen saturation in a burn wound under the action of BC-ZnO NPs-BDP and a decrease in hypoxia. In turn, the enhancement of microcirculation in burn wounds acts not only on the blood flow rate in arterioles, capillaries, venules, and other vessels, but is also able to influence the blood coagulation system under hypoxia. An improvement in oxygen saturation due to a regulatory mechanism—respiratory control—is also associated with the increased oxidation of substrates (fatty acids, glycolysis products, and participants in the Krebs cycle). The oxidation of substrates is mainly regulated by allosteric mechanisms and is most consistently stimulated by an excess of ADP [47]. In addition, oxidative phosphorylation also depends on the presence of O_2_, NADH, ADP, and the phosphate residue Pi in the substrate and controls the rate of this process. For example, severe hypoxia or vascular occlusion restricts oxygen availability and can accelerate cell death. Such oxygen starvation reflexively increases the concentration of ADP and NADH while the concentration of ATP decreases.

The introduction of ADP into platelet-rich plasma (PRP) in the presence of the studied antiplatelet agents (BDP, ZnO NPs-BDP, and ASA) causes not only platelet aggregation but also causes the process of platelet disaggregation (Table 2 and Figure 2, Table 3 and Figure 3).

These results can be explained by the contribution of charge–charge interactions between the ADP agonist and BDP immobilized in BC and ZnO NPs. To substantiate the charge-charge interactions, we carried out electrokinetic studies to measure the zeta potential and hydrodynamic diameter of wound dressings component ZnO NPs, modified with compounds with phosphate groups. We used a BDP solution in a phosphate buffer solution at pH 7.44, in which phosphate groups are ionized and exist partially in the form of sodium salts. For comparison, we carried out studies in a solution containing phytic acid and sodium phytate.

The zeta potential of ZnO NPs (sizes are equal to 12–20 nm according to PXRD data) in distilled water is +15.9 ± 1.20 mV. From the data in Table 8, it follows that ZnO NPs in aqueous solutions of BDP (water or phosphate buffer solution with pH 7.20–7.44) has a negative charge and a large hydrodynamic diameter, which makes it possible to stabilize the ZnO NPs-BDP colloidal system without additional stabilizers.

**Figure 4:**
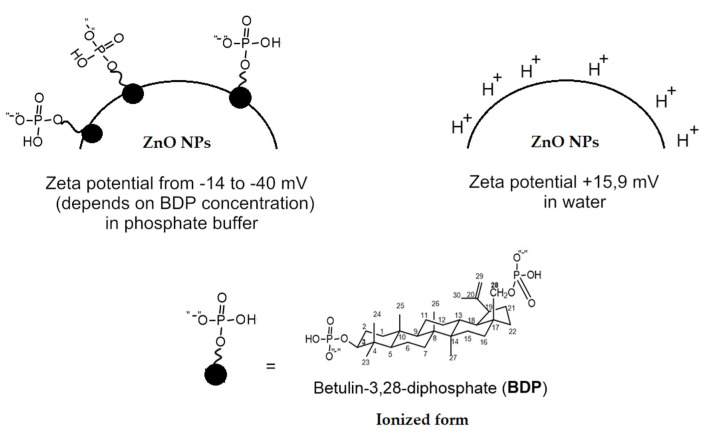
Schematic illustration of BDP ionization in phosphate-buffered saline, BDP immobilized into ZnO NPs and ZnO NPs in water.

The negative charge characterizes the ZnO particle with adsorbed BDP in ionized form with a phosphate ion group (Figure 4).

The reversibility of disaggregation–aggregation under the action of ADP in the BDP and ZnO NPs-BDP system, as well as phytic acid and sodium phytate, is probably due to the appearance of AMP and changes in the phosphate pool in the system, including the ionization (degree of ionization) of phosphate groups. Thus, the authors of [48] showed that the sodium salt of phytic acid, which has an extremely high charge density of 12 anionic charges per inositol moiety, demonstrated strong anticoagulant activity using in vitro and in vivo experiments (on rats and healthy volunteers). In contrast, phytic acid increased the size of aggregates under the action of agonists [49]. The authors of [50] suggested that inhibition of agonist-induced platelet aggregation promoted the treatment of oncological diseases by the example of colon cancer. We can assume that the inhibition of aggregation under the action of BDP and ZnO NPs-BDP, in which phosphate groups are ionized, will promote the healing of burn wounds.

**Figure 5:**
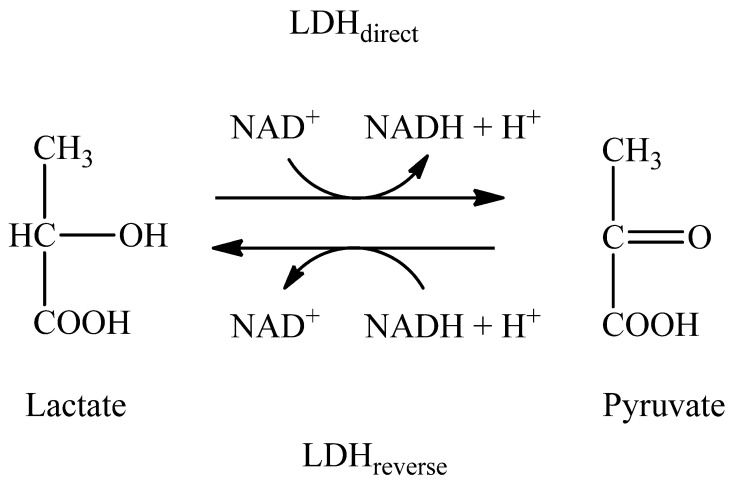
Mechanism of the action of lactate dehydrogenase in direct and reverse reactions.

The interruption of blood microcirculation in a burn wound, leading to a lack of oxygen (hypoxia), causes a change in the ratio of LDH activity in the direct and reverse reactions (Table 4), and, accordingly, leads to an imbalance in the pairs of coenzymes NAD+/NADH.

l-lactate dehydrogenase converts lactate in a direct reaction to pyruvate, which is then used in the Krebs cycle. NAD+ is regenerated by converting pyruvate to lactate (reverse reaction) (Figure 5).

In most tissues, the total concentration of [NAD+] + [NADH] is ~10^−5^ M, while the ratio NAD+(oxidation)/NADH(reduction) is large, which favors the transfer of the hydride ion from the substrate to NAD+ with the formation of NADH.

From the data of [2], it follows that with burns, the NADH concentration changes insignificantly (within 7 days after the burn), “−”0.012–0.014 nmol/mg wet weight, which is a percentage of 8.4 (12 h) to 11.9 (7 days) from the total concentration of [NAD+]+[NADH]. For intact animals, the NADH value was 18.2%.

Our data on animals with burns without treatment are consistent with work [2] on the measurement of the concentration of NAD+ during the burn disease in an in vitro experiment on mice. In the study [2], the concentration of NAD+ on Day 3 decreased almost twice with the burn disease without treatment. On Day 7, the NAD+ concentration decreased almost four times compared with the initial state of the burn: from 0.374 to 0.167 nmol/mg wet weight (Day 3) and up to 0.089 nmol/mg wet weight (Day 7).

When treated with Oleogel BC-BDP, BC-ZnO, and NPs-BDP, the ratio of LDH activity ($\frac{{\mathrm{LDH}}_{\mathrm{reverse}}}{{\mathrm{LDH}}_{\mathrm{direct}}}$) on Day 3 increased compared to the burn to 8.70, which indirectly characterizes the increase in the concentration of NAD+, which is required for obtaining pyruvate and its further participation in the Krebs cycle. With further treatment, this value decreased slightly and became close to the norm (7.60).

Therefore, the empirical assessment of the ratio of LDH activity ($\frac{{\mathrm{LDH}}_{\mathrm{reverse}}}{{\mathrm{LDH}}_{\mathrm{direct}}}$) can be used as a predictor of the course of burn disease and the effectiveness of burn treatment.

The level of cellular NAD(H)/NADP(H) is essential for maintaining redox homeostasis. Deficiency in these redox couples can lead to oxidative or reductive stress, depending on the redox ratio of each. Both oxidative stress and reductive stress are detrimental to normal cell functions. This dual role complicates the use of global antioxidants as rational and effective therapeutic approaches to redox stress disorders. An imbalance of these two redox couples directly affects energy metabolism. This imbalance alters NAD(H)/NADP(H)-dependent enzymes and, thus, affects their functions in regulating cellular metabolism [4].

The increase in GR activity under the treatment by BC-ZnO NPs-BDP in comparison with the untreated burnt group leads to an increase in the fraction of antioxidants glutathione (GSH) and NADPH in cells.

NADPH and glutathione serve as dual-function participants in maintaining cellular redox homeostasis. GSH is a cosubstrate (antioxidant) for hydrogen peroxide (H_2_O_2_) removal by glutathione peroxidases (GPxs). NADPH functions as an indispensable cofactor for glutathione reductase (GR) that is essential for GPx-mediated peroxide removal. GR catalyzes the recycling of GSH from its oxidized form (GSSG). Excess accumulation of GSH and/or NADPH leads to reductive stress, and may directly contribute to the production of “O^2−”^ and H_2_O_2_.

It can be proposed that ionizable phosphate groups of BDP regulate the NADPH functions of GR.

Another principal source of cytosolic NADPH is G6PDH. It affects cellular NADPH levels and consequently the cellular redox state and biological functions.

Data of Table 6 on the significant increase in G6PDH activity under the treatment by BC-ZnO NPs, BC-BDP, and BC-ZnO NPs-BDP indirectly characterize the increase in cytosolic NADPH concentration.

It is difficult to assess the effect of wound dressings components (BDP or ZnO NPs) on improving biochemical indexes such as the activity of the enzymes GR and G6PDH.

One of reasons for GR and G6PDH activation can be the ability of BDP to contribute to the phosphate pool in the biosystem.

## 4. Materials and Methods

### 4.1. Preparation of BC

BC was produced using previously described methods [29,51]. In a typical preparation, BC was obtained in a static culture medium by *Komagataeibacter sucrofermentans* H-110, which was isolated from Kombucha tea and identified by sequencing the amplified product of 16S rRNA. For the production of BC, a Hestrin and Schramm (HS) medium that contained glucose (20 g∙L^−1^), peptone (5 g∙L^−1^), yeast extract (5 g∙L^−1^), citric acid (1.15 g∙L^−1^), and disodium hydrogen phosphate (2.7 g∙L^−1^) at a pH of 6.0 was used. The culture medium was autoclaved and inoculated with 10% (*v*/*v*) inoculum. After incubation at 28 °C for 5 days, the BC was collected, washed, and the bacterial cells were removed. The ^13^C nuclear magnetic resonance (NMR) spectrum of dried BC used in this study is a typical spectrum of the BC crystal structure [29].

### 4.2. Betulin-3,28-diphosphate

Betulin-3,28-diphosphate (BDP, 3β,28-diphosphate-lup-20(29)-ene) was synthesized according to the procedure [52], and its properties correspond to the literature data [52].

### 4.3. Zinc Oxide Nanoparticles

Two methods of ZnO NPs preparation were used in this work.

(1) ZnO NPs were prepared by sol–gel methods [53]; modified methods were described in our work [29] previously.

(2) ZnO NPs-PEG were obtained using methods in [29,54]. All properties (FTIR, PXRD patterns, and SEM images) are shown in Figure S2, and they correspond to literature data [29].

### 4.4. Oleogel ZnO NPs-BDP

Oleogel ZnO NPs-BDP preparation and properties were described in detail in [55]. Formulation of oleogel, %: ZnO NPs (5.0), BDP (1.0), and sunflower oil up to 100.0.

### 4.5. Preparation of BC-ZnO NPs-BDP Composites and Their Properties

The BC film was placed in aqueous alcohol (1:1) solutions containing BDP∙11.5H_2_O (1%) at pH 7.44 for swelling for 30 min. After swelling, a suspension of an aqueous-alcoholic solution of zinc oxide nanoparticles modified by BDP was sprayed onto the surface of an elastic polymer mesh at the weight ratio of ZnO NPs to initial BC film equal to 5:100. After removing the excess solvent by vacuum drying, the films were closed on both sides with parchment and sealed in cellophane envelopes.

When treating rats with BC-ZnO NPs, BC-BDP, and BC-ZnO NPs-BDP composite coatings, the composite coatings were additionally moistened with 0.9% sodium chloride solution in the presence of 0.01% lidocaine and 0.01% benzalkonium chloride.

### 4.6. FTIR Analysis

FTIR spectra in the 400–4000 cm^−1^ range were measured by an IR Prestige-21 FTIR spectrometer (Shimadzu, Kyoto, Japan) equipped with a KBr beam splitter. A pellet from a well-dried KBr was prepared according to standard cold pressing. The resolution was 0.5 cm^−1^, and the number of scans was 45.

### 4.7. UV Analysis

UV spectra were recorded by UV-1800 (Shimadzu, Kyoto, Japan).

### 4.8. RP-HPLC Analysis

Reversed phase high-performance liquid chromatography analysis was carried out on an LC-20Avp (Shimadzu, Kyoto, Japan) with UV-detection, and the column was a Discovery C18 (25 cm × 4.6 mm, 5 μm, Supelco).

### 4.9. Specific Surface Areas Analysis

Specific surface areas (SSAs) of the ZnO NPs were analyzed using an analyzer of specific surface area and adsorption porosity ASAP 2020 (Micromeritics, Norcross, GA, USA).

### 4.10. Chemical Composition of BC

The chemical composition of BC was analyzed by determining C/O/N using a Flash EA 1112 CN analyzer (NEOLAB Ltd., VELLETRI, ROMA, Italy). Analysis for major and trace elements was performed with an atomic absorption spectrophotometer AA-7000 (Shimadzu, Kyoto, Japan) after wet mineralization of cellulose samples with a mixture of perchloric and nitric acids.

### 4.11. Surface Charge and Dynamic Light Scattering Measurements

The surface charge and average hydrodynamic diameter of the ZnO NPs were measured using NanoBrook Omni (Brookhaven Instruments, NY, USA). Suspensions with a solid loading of 0.00625–0.02500% were prepared in the presence (5 × 10^−4^ M) of BDP in different mediums and were allowed to equilibrate for 24 h to reach the steady state. The zeta potential was determined by phase analysis electrophoretic light scattering (PALS). The Smoluchowski model was used to convert the electrophoretic mobility values to the zeta potential values.

The hydrodynamic diameter of ZnO nanoparticles was determined by dynamic light scattering (DLS) in the mode of multimodal analysis of the correlation function. The measurements were carried out at 25 ± 0.1 °C at an angle of 90° in the range from 0.1 to 5000 nm in polystyrene cuvettes (1 cm). The accumulation time of the correlation function was 180 s (*n* = 10).

### 4.12. Biological Activity

Male Wistar rats (150–200 g) were involved in the study. The animals were purchased from the Animal Breeding Facilities “Andreevka” Federal State Budgetary Institution of Science “Scientific Center for Biomedical Technologies” of the Federal Medical and Biological Agency (Andreevka, Moscow region, Russia). The animals were handled humanely, kept in plastic suspended cages, and placed in a well-ventilated and hygienic rat house under suitable conditions of room temperature (27 ± 2 °C) and humidity. They were given food and water ad libitum and subjected to a natural photoperiod cycle of 12 h light and 12 h dark. The animals were allowed two weeks of acclimatization before the commencement of all animal model experiments in the study.

All blood taking and withdrawal of the animals for the experiment were performed under anesthesia, all efforts being made to minimize suffering.

The animal study was conducted according to the guidelines of the Declaration of Helsinki, and approved by the Local Ethics Committee of Privolzhsky Research Medical University, Russian Federation (protocol No. 1 from 18 January 2021).

#### 4.12.1. Modeling of Thermal Burns in Animals

The deep second-degree thermal burns of the rat’s back was made using electromagnetic radiation from an infrared soldering station YaXunXY865D following the requirements of Good Laboratory Practice for the experimental modeling of thermal burns in laboratory animals. Standard thermal burns had an area of 14.0 ± 0.5 cm^2^, and the average weight of the rats was 285.0 ± 5.0 g.

#### 4.12.2. Microcirculation Research

Microcirculation was assessed quantitatively using the LAKK-02 (LASMA, Moscow, Russia). This device transmits continuous-wave laser light (30 mW, 890 nm) and white light (20 W, 500–900 nm) to skin tissue near the wound, where it is scattered and collected on the skin surface with fibers of the probe. The movement of erythrocytes causes a Doppler shift, which, in turn, is detected by the laser light and analyzed by the LAKK-02, which is then computed and displayed as the blood flow velocity. The detected laser signal correlates with the number of moving erythrocytes in tissue and blood flow velocity for calculation of the microcirculation parameters, using such arbitrary (relative) units (arb. un.) as perfusion units (perf. un.). The rate of microcirculation (the microcirculation level), the regulatory activity of its components, and the degree of shunt path participation with an allowance for the frequency range intervals of the blood flow oscillations in the rats’ microvessels were investigated [56,57].

#### 4.12.3. Biological Analysis In Vitro

In vitro biological analysis was performed using blood stabilized with sodium citrate. Erythrocytes were washed twice with 0.9% NaCl by centrifugation for 10 min at 1600× *g*. Glutathione reductase activity (EC 1.8.1.7) was studied by spectrophotometry based on the oxidized glutathione reduction [58]. The activity of glucose-6-phosphate dehydrogenase (EC 1.1.1.49) was determined in hemolysate of erythrocytes by spectrophotometry based on glucose-6-phosphate oxidation to the phosphoglucolactone with the formation of reduced nicotinamide adenine dinucleotide phosphate (NADPH) [59]. The energy metabolism in erythrocytes was studied using the catalytic activity of LDH (LDH, EC 1.1.1.27), directly (LDH_direct_, substrate—50 mM of sodium lactate) and in reverse (LDH_reverse_, substrate—23 mM of sodium pyruvate) reactions [60]. The activity of aldehyde dehydrogenase (EC 1.2.1.3) was estimated spectrophotometrically in accordance with previous methods [61]. The specific activity of the enzymes was calculated from the protein concentration analyzed by the modified Lowry method [62].

#### 4.12.4. Platelet-Rich Plasma (PRP) and Platelet-Poor Plasma (PPP) Preparation

This study was performed in human platelets from 10 healthy volunteers aged 18 to 50 years, which were free of medication for 7 days. Volunteers signed the informed consent before participating in this study. This study was approved by the Local Ethics Committee of Privolzhsky Research Medical University, Russian Federation (protocol No. 1 from 18 January 2021). Blood samples (30 mL) from overnight fasting healthy volunteers were collected by venipuncture and put into plastic tubes containing 3.2% sodium citrate (blood:buffer = 9:1 *v/v*). The platelet-rich plasma (PRP) was prepared by centrifugation of blood samples at 21 °C, 200× *g* (1300 rpm) for 7 min, and the top layer was collected as PRP. Isolation of the platelet-poor plasma (PPP) was performed by further centrifugation of the rest of the blood samples at 21 °C, 2000× *g* (4000 rpm) for 15 min. PPP was used as a reference to define the theoretical point of 100% light transmission. Aggregation testing was performed at least 15 min after PRP was prepared to recover from refractoriness and used within 4 h of sample collection.

#### 4.12.5. Platelet Aggregation Measurement

The platelet aggregation test was performed in accordance with [63], which was modified from the Born turbidimetric aggregometry method [45] using an aggregometer ALAT-2 laser aggregation analyzer (BIOLA, Moscow, Russia). PRP (300 µL) was set as 0% light transmission. PPP (300 µL) was set as 100% light transmission. We recorded changes in the light transmittance (at 785 nm wavelength) for 5 min after adding 15 µL of ADP solution. The submaximal concentration of ADP (the final concentration was equal to 3.75 µM) was used to induce platelet aggregation. Two minutes after the start of the experiment, 100 µL of BDP solution or ZnO NPs-BDP suspension was added to the studied solution. Acetylsalicylic acid (0.1 mM) was used as a positive control. PRP and all used solutions were pre-incubated for 5 min at 37 °C. Each sample was analyzed in triplicate. The light transmission dependence on time corresponding to the dependence of the percentage of platelet aggregation on time was presented.

### 4.13. Statistical Analysis

Statistical data processing was performed by Statistica 6.0 software (StatSoft Inc., Tulsa, OK, USA). The normality of the distribution of results was shown using the Shapiro–Wilk test. The significance of differences between groups was assessed using Student’s t-test and one-way analysis of variance (ANOVA). The differences were considered statistically significant at *p* < 0.05.

## 5. Conclusions

It has been shown that BDP and ZnO NPs-BDP can inhibit aggregation due to ionized phosphate groups in BDP. This anti-aggregation effect promotes the healing of burn wounds. To inhibit ADP-induced platelet aggregation in plasma under the action of betulin diphosphate, it is important to ionize its two phosphate groups, which are potential donors of phosphoryl groups and can affect the total phosphate pool. This ability to inhibit platelet aggregation is retained upon introducing BDP into the bacterial cellulose matrix or upon immobilization in ZnO NPs. The antiaggregatory effect can be enhanced by bacterial cellulose in polyanionic form. We can assume that polyanionic compounds with a high charge, with ionized phosphate or phosphoryl groups, will be advantageous as antiplatelet agents.

The work demonstrates the high enzymatic activity of oxidoreductases (LDH, AlDH, GR, and G6PDH) under conditions of hypoxia caused by a burn in the treatment of wound dressings based on bacterial cellulose and zinc oxide nanoparticles with betulin diphosphate. The ratio of specific LDH activity in reverse and direct reactions $\frac{{\mathrm{LDH}}_{\mathrm{reverse}}}{{\mathrm{LDH}}_{\mathrm{direct}}}$ can be used as an empirical assessment of NAD+/NADH imbalance in the development of burn disease and a predictor of the effectiveness of burn treatment.

## Figures and Tables

**Table 1:** Data of the wavelet-LDF spectra obtained on the wound on Day 10 of treatment (Figure 1) ^1^.

System	Microhemocirculation Properties			
	R/E Ratio	R/N Ratio	R/C Ratio	MI, Perf. un.
Burn (Day 0)	0.79	0.52	2.73	6.31 ± 0.57
Burn (Day 10)	0.12	0.20	1.14	11.94 ± 0.71
Oleogel ZnO-BDP	6.00	3.43	6.86	13.19 ± 1.20
BC-ZnO	2.88	1.92	6.57	12.96 ± 1.97
BC-ZnO-BDP	18.67	16.0	3.86	14.86 ± 1.35
BC-ZnO-BDP (Day 21)	17.58	22.57	7.18	13.26 ± 1.21
Intact	18.25	24.33	5.62	13.58 ± 1.62

^1^ M.I. (burned on Day 3) = 11.51 ± 0.94 perf. un.

**Table 2:** The inhibition (%) of platelet aggregation by 6.65 mM of BDP, ZnO NPs-BDP 6.65 mM, and ASA 1.40 mM.

Agent	I Stage (Disaggregation–Aggregation)		II Stage (Disaggregation–Aggregation)	
	% Inhibition (max)	Time (min)	% Inhibition (max)	Time (min)
BDP 6.65 mM	58	2.71	90	5.00
ZnO NPs-BDP 6.65 mM	29	2.63	90	4.62
ASA 1.40 mM	38	2.51	90	4.71

**Table 3:** The inhibition (%) of platelet aggregation under the action of various concentrations of substances.

Sample	Concentration	% Inhibition (4 min)	Max. Inhibition	
			% Inhibition (max)	Time (min)
BDP	4.43 mM	73	90	5.00
	2.22 mM	9	85	3.17
	0.44 mM	53	55	3.87
ZnO NPs (11–20 nm)	0.01%	0	1	4.12
	0.005%	0	3	3.98
	0.001%	0	4	4.20
ZnO NPs-BDP	0.01%–4.43 mM	65	86	4.83
	0.005%–2.22 mM	31	83	4.72
	0.001%–0.44 mM	38	46	3.56

**Table 4:** The specific activity of lactate dehydrogenase in direct and reverse reactions under the action of the studied drugs (% of control), *n* = 3, *p* < 0.001.

Enzyme	τ, Day	Enzyme Activity, % of Control			
		Burnt (Untreated)	Oleogel	BC-BDP	BC-ZnO NPs-BDP
LDH_direct_ ^1^	3	66.89 ± 4.21	81.70 ± 5.50	92.15 ± 3.16	96.21 ± 4.40
	7	86.54 ± 4.26	101.58 ± 8.50	102.76 ± 1.03	110.67 ± 1.40
	10	96.43 ± 6.06	112.59 ± 6.02	118.28 ± 1.17	126.26 ± 1.73
	21	N/a	111.97 ± 5.78	129.57 ± 2.37	138.25 ± 2.31
LDH_reverse_ ^2^	3	57.73 ± 1.54	88.91 ± 3.82	82.34 ± 12.56	110.05 ± 5.99
	7	60.26 ± 2.12	93.89 ± 2.86	96.36 ± 2.68	105.82 ± 2.36
	10	72.72 ± 2.98	98.82 ± 3.70	101.90 ± 2.23	108.28 ± 3.55
	21	N/a	96.10 ± 3.22	107.65 ± 4.15	123.71 ± 3.79

^1^ LDH_direct_ 100% − 22.772 ± 1.265 nmol NAD^+^ min^−1^ mg protein^−1^ (Intact group); ^2^ LDH_reverse_ 100% − 173.179 ± 4.368 nmol NADH min^−1^ mg protein^−1^ (Intact group).

**Table 5:** Data of the $\frac{{\mathrm{LDH}}_{\mathrm{reverse}}}{{\mathrm{LDH}}_{\mathrm{direct}}}$ ratio, calculated using data of Table 4 ^1^.

τ, Day	Burnt (Untreated)	Oleogel	BC-BDP	BC-ZnO NPs-BDP
3	6.56	8.28	6.80	8.70
7	5.30	7.03	7.13	7.27
10	5.74	6.67	6.55	6.52
21	N/a	6.53	6.32	6.81

^1^ Intact ratio is equal to 7.60.

**Table 6:** The activity of aldehyde dehydrogenase under the action of the studied drugs (% of control), *n* = 3, *p* < 0.001.

τ, Day	AlDH Activity, % of Control ^1^			
	Burnt (Untreated)	Oleogel	BC-BDP	BC-ZnO NPs-BDP
3	49.05 ± 0.38	140.46 ± 1.78	139.85 ± 2.43	146.88 ± 2.44
7	48.58 ± 0.43	148.57 ± 1.80	179.77 ± 2.64	279.21 ± 1.80
10	58.19 ± 2.64	138.61 ± 1.13	172.09 ± 2.31	115.00 ± 4.62
21	N/a	109.18 ± 3.41	119.54 ± 2.00	101.53 ± 0.97

^1^ 100% − 22.359 ± 0.947 nmol NAD^+^ min^−1^ mg protein^−1^ (Intact group).

**Table 7:** The activity of glutathione reductase and glucose-6-phosphate dehydrogenase under the action of the studied drugs (% of control), *n* = 3, *p* <0.001.

Enzyme	τ, Day	Enzyme Activity, % of Control			
		Burnt (Untreated)	Oleogel	BC-BDP	BC-ZnO NPs-BDP
GR ^1^	3	54.04 ± 1.07	78.99 ± 2.06	85.61 ± 3.63	110.35 ± 6.26
	7	62.94 ± 1.16	81.61 ± 4.26	87.60 ± 3.56	90.53 ± 2.89
	10	70.29 ± 1.04	89.47 ± 2.72	87.49 ± 2.35	99.45 ± 1.45
	21	N/a	91.52 ± 2.29	88.97 ± 0.83	115.36 ± 2.17
G6PDH ^2^	3	80.24 ± 1.48	107.15 ± 4.14	97.62 ± 1.90	134.89 ± 2.74
	7	93.28 ± 0.45	123.80 ± 2.74	139.77 ± 1.72	164.20 ± 5.14
	10	103.55 ± 0.95	125.87 ± 2.92	146.72 ± 2.90	173.19 ± 3.92
	21	N/a	136.40 ± 4.45	142.85 ± 1.45	191.04 ± 6.82

^1^ GR 100% − 96.527 ± 3.178 nmol NADPH min^−1^ mg protein^−1^ (Intact group); ^2^ G6PDH 100% − 27.863 ± 0.464 nmol NADPH min^−1^ mg protein^−1^ (Intact group).

**Table 8:** Data of zeta potential and hydrodynamic diameter of ZnO NPs dispersions (20 mg/10 mL) in solutions of 2.66∙10^−2^ M BDP.

Medium	pH	Zeta Potential, mV	Hydrodynamic Diameter (Multimodal Mode) ^1^
BDP aqueous solution	7.44	−7.75 ± 1.74	80–130; 300–400
BDP in phosphate buffer	7.20	−14.53 ± 1.34	60–100; 350–450
Sodium salt of phytic acid in phosphate buffer	7.20	−13.88 ± 3.00	450–800; 3000–4500; 40, 60, 130
H_2_O	5.80	+15.9 ± 1.20	N/a

^1^ The hydrodynamic diameter was calculated by DLS based on scattering intensity.

## Supplementary Materials

The following are available online, Figure S1: Images of intact (a) and burnt rats without treatment (Day 0, b and Day 10, c), and under the treatment of BC-BDP (d, g), BC-ZnO NPs (e, h), and BC-ZnO NPs-BDP (f, i), Table S1: The decrease in wound area in % of an initial burn wound state during the treatment, Figure S2: Properties of ZnO NPs: powder XRD pattern (a), SEM image (b), and FTIR-spectrum (c).

## Sample Availability

Samples of the compounds BDP, ZnO NPs, and BC are available from the authors.
