# Supplementary material for: The Effect of Betulin Diphosphate in Wound Dressings of Bacterial Cellulose-ZnO NPs on Platelet Aggregation and the Activity of Oxidoreductases Regulated by NAD(P)+/NAD(P)H-Balance in Burns on Rats
Source: Molecules. 2021 Sep 9;26(18):5478. doi: 10.3390/molecules26185478 (PMC8469126; doi:10.3390/molecules26185478)
Supplement: Supplementary file 1 [file molecules-26-05478-s001.zip › molecules-1352171-supplementary.pdf]

Supplementary materials

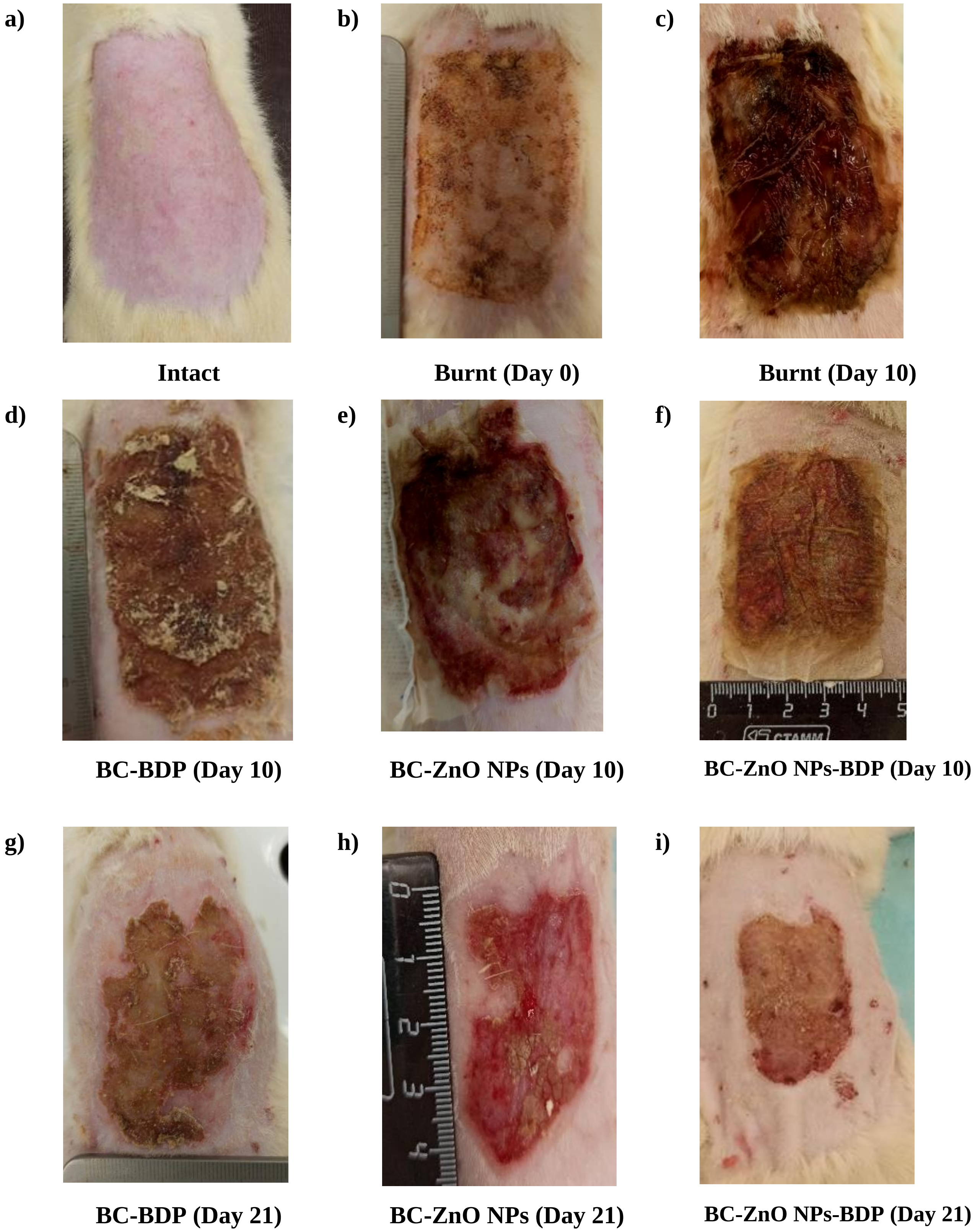

Figure S1. Images of intact (a) and burnt rats without treatment (Day 0, b and Day 10, c), and under the treatment of BC-BDP (d, g), BC-ZnO NPs (e, h) and BC-ZnO NPs-BDP (f, i).

Table S1. The decrease of wound area in % of an initial burn wound state during the treatment [1].

Treatment (group)	The decrease of wound area, %	
	Day 10	Day 21
BC-BDP	8.45	38.41
BC-ZnO NPs	9.71	39.18
BC-ZnO NPs-BDP	10.33	42.31

[1] Initial burn wound area is 21.65±0.98 cm^2 taken as 100%

a)

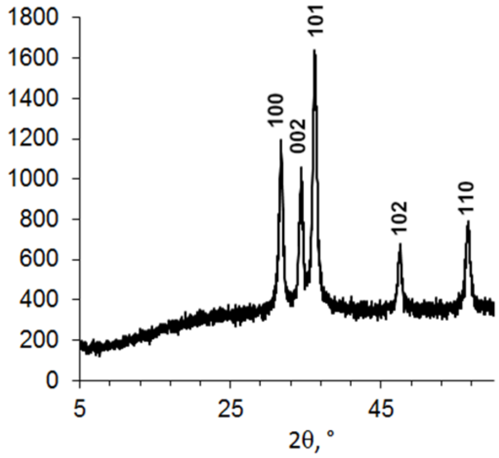

b)

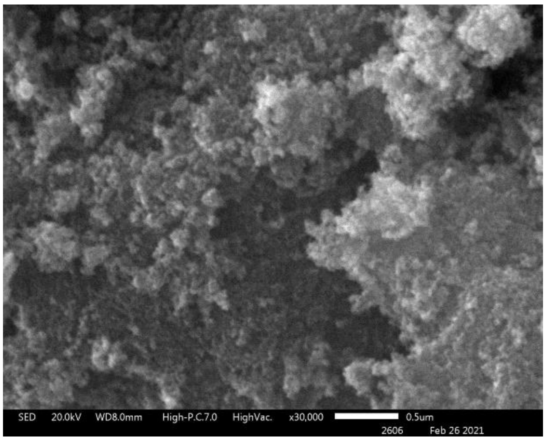

Composition (treatment [1])	2θ, degree	D, nm
	36.18	14.040
ZnO NPs-PEG	47.60	11.562
	56.54	12.285

c)

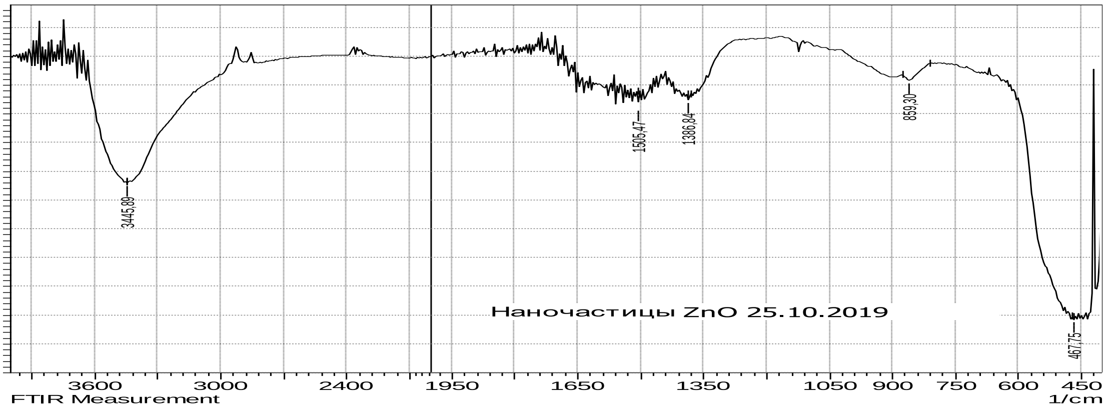

Figure S2. Properties of ZnO NPs: powder XRD pattern (a), SEM image (b), FTIR-spectrum (c).
